# Non‐structural protein 1‐specific antibodies directed against Zika virus in humans mediate antibody‐dependent cellular cytotoxicity

**DOI:** 10.1111/imm.13380

**Published:** 2021-06-14

**Authors:** Luis A. Sanchez Vargas, Awadalkareem Adam, Mary Masterson, Madison Smith, Zoe L. Lyski, Kimberly A. Dowd, Theodore C. Pierson, William B. Messer, Jeffrey R. Currier, Anuja Mathew

**Affiliations:** ^1^ Department of Cell and Molecular Biology Institute for Immunology and Informatics University of Rhode Island Providence RI USA; ^2^ Viral Diseases Branch Walter Reed Army Institute of Research Silver Spring MD USA; ^3^ Department of Molecular Microbiology and Immunology Oregon Health & Science University Portland OR USA; ^4^ Laboratory of Viral Diseases NIAID, NIH Bethesda MD USA; ^5^ Division of Infectious Diseases Department of Medicine Oregon Health & Science University Portland OR USA; ^6^ OHSU‐PSU School of Public Health Program in Epidemiology Oregon Health & Science University Portland OR USA

**Keywords:** antibody‐dependent cellular cytotoxicity, B cell, FluoroSpot, humans, immunity, non‐structural protein 1, Zika virus

## Abstract

There is growing interest in understanding antibody (Ab) function beyond neutralization. The non‐structural protein 1 (NS1) of Zika virus (ZIKV) is an attractive candidate for an effective vaccine as Abs against NS1, unlike the envelope or premembrane, do not carry the risk of mediating antibody‐dependent enhancement. Our aim was to evaluate whether ZIKV NS1 Abs elicited following natural infection in humans can mediate antibody‐dependent cellular cytotoxicity (ADCC). We evaluated the isotype specificity of ZIKV‐specific Abs in immune sera and supernatants from stimulated immune PBMC and found that Abs against ZIKV NS1 and virus‐like particles were predominantly of the IgG1 isotype. Using a recently developed FluoroSpot assay, we found robust frequencies of NS1‐specific Ab‐secreting cells in PBMC of individuals who were naturally infected with ZIKV. We developed assays to measure both natural killer cell activation by flow cytometry and target cell lysis of ZIKV NS1‐expressing cells using an image cytometry assay in the presence of ZIKV NS1 Abs. Our data indicate efficient opsonization of ZIKV NS1‐expressing CEM‐NK^R^ cell lines using ZIKV‐immune but not ZIKV‐naïve sera, a prerequisite of ADCC. Furthermore, sera from immune donors were able to induce both NK cell degranulation and lysis of ZIKV NS1 CEM‐NK^R^ cells in vitro. Our data suggest that ADCC is a possible mechanism for ZIKV NS1 Abs to eliminate virally infected target cells.

## Graphical abstract

ZIKV‐immune sera efficiently opsonize CEM‐NKR cell lines expressing ZIKV NS1. Sera from immune donors can induce both NK cell degranulation and lysis of ZIKV NS1 CEM‐NKR cells in vitro.

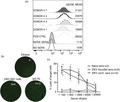

**Abbreviations**
ADCC: antibody‐dependent cellular cytotoxicity
ASCs: antibody‐secreting B cells
DENV: dengue virus
FL‐ZIKV: fluorescently labeled Zika virus
IAV: influenza virus
MBCs: memory B cells
NS1: non‐structural protein 1
PBMC: peripheral blood mononuclear cell
VLPs: virus‐like particles
ZIKV: Zika virus

## INTRODUCTION

Zika virus (ZIKV) is a ssRNA virus of the Flaviviridae family that includes dengue virus (DENV), West Nile virus (WNV) and yellow fever virus (YFV) [1, 2]. Given the co‐circulation of DENV and ZIKV in endemic countries, a significant amount of effort has been spent to understand the landscape of Abs and T cells elicited in response to ZIKV and cross‐reactivity with DENV [3, 4, 5]. Prospective studies in endemic countries suggest that prior infection with DENV protects from symptomatic ZIKV infection but prior infection with ZIKV may actually enhance the future risk of severe infection with DENV [5, 6, 7].

Abs against the structural proteins of flaviviruses, envelope (E) and premembrane (prM), have the potential to exacerbate disease with the same or related flavivirus through antibody‐dependent enhancement (ADE) [8, 9]. A large outbreak in central and South America in 2015–16 brought to light some of the unique characteristics of symptomatic ZIKV infection, which included birth defects such as microcephaly in infants born to pregnant mothers infected during the first or second trimester [10, 11, 12, 13]. In animal models, prior immunity with DENV enhanced replication in ZIKV‐infected pregnant mice, and significantly increased placental damage, fetal growth restriction and fetal resorption [14]. The mechanism was FcγR‐mediated and supported concerns that Abs against structural proteins of flaviviruses might worsen disease.

The non‐structural protein 1 (NS1) of flaviviruses is a complex glycoprotein involved in viral replication, immune evasion and immunopathogenesis [15]. DENV NS1 can directly stimulate inflammatory cytokine secretion from endothelial cells. DENV and ZIKV NS1 can also alter the barrier function of pulmonary endothelial cell monolayers through disruption of the endothelial glycocalyx‐like layer [16, 17, 18]. NS1 Abs against YFV and WNV are known to activate Fc‐mediated effector functions antibody‐dependent cellular cytotoxicity (ADCC), antibody‐dependent cellular phagocytosis (ADCP) and antibody‐dependent complement deposition (ADCD) [19, 20]. NS1mAbs were able to protect against a lethal DENV‐2 challenge in IFN‐deficient mice [21]. A number of human and mouse mAbs against ZIKV NS1 protected against ZIKV challenge in both non‐pregnant and pregnant mice through Fc effector functions [22, 23]. Two recent papers assessed the mechanistic and structural basis of flavivirus NS1‐specific mAbs in blocking permeability in cell lines, reducing viraemia in murine models, and inhibition of NS1 induced endothelial cell dysfunction [24, 25].

Sharma et al. [26] found that polyclonal sera post‐vaccination from a phase 2 clinical trial of Takeda's live attenuated tetravalent dengue vaccine candidate inhibited DENV‐2 NS1‐induced hyperpermeability and the degradation of endothelial glycocalyx components. ADCC activity is known to correlate with protection against several viral infections and the efficacy of experimental vaccines [27]. Recently, Abs in ZIKV‐immune but not ZIKV‐naïve sera, against ZIKV prM and E proteins, were found to mediate ADCC [28]. The function of ZIKV NS1‐specific Abs following natural infection or vaccination in humans is less well understood. Furthermore, the magnitude of NS1‐specific memory B cells (MBCs) in convalescent PBMC and isotype specificity of Abs encoded by these MBCs has not been characterized.

In the current study, we characterized the specificity and magnitude of memory B cells and measured the isotype specificity of ZIKV NS1 Abs in sera and supernatants from stimulated PBMC, obtained up to one year post‐natural ZIKV infection. We next developed and optimized assays to assess the ADCC activity of ZIKV NS1 sera by measuring both NK cell activation and target cell lysis. Using CEM‐NK^R^ cell lines stably transfected with ZIKV NS1, we show immune but not naïve sera induce NK cells to become activated, degranulate and mediate target cell lysis. This is the first indication that Abs generated against ZIKV NS1 following natural viral infection are functional as measured by in vitro ADCC assays.

## MATERIALS AND METHODS

### Samples

PBMCs from 12 ZIKV‐immune donors were used in the study (Table 1). All ZIKV PBMCs were collected after written informed consent. The studies were approved by the institutional review boards at the University of Rhode Island, National Institute of Allergy and Infectious Diseases (NIAID) and Oregon Health & Science University (OHSU). Blood samples (Donors 1–8) of travellers were collected at the National Institutes of Health Clinical Center from patients displaying symptoms of a suspected ZIKV infection following return to the United States from areas where ZIKV was known to be circulating. Donors 9–12 were enrolled in a flavivirus immune cohort at Oregon Health & Science University [29]. PBMCs were isolated and cryopreserved prior to use. ZIKV‐immune sera or plasma (*n* = 28) were obtained from BEI resources and OHSU. The ZIKV AccuSet™ plasma performance panel was purchased from SeraCare Life Sciences (Milford, MA).

**TABLE 1:** ZIKV convalescent PBMC and infection history

Donor ID	Confirmation of ZIKA infection	Approximate days after infection	Previous flavivirus history	ZIKV NT_50_ titres
1^a^	PCR, urine	138 days	YFV vaccine 2002	1:1695
2^a^	PCR, urine	52 days	None	1:3326·5
3^a^	PCR, urine	68 days	YFV vaccine 2007	1:5481·5
4^a^	PCR, urine	66 days	None	1:2950
5^a^	PCR, urine	14 days	YFV vaccine 2002	1:28 907
6^a^	PCR, urine	43 days	None	1:3223·5
7^a^	PCR, urine	24 days	None	1:32 485·5
8^a^	PCR, urine	41 days	None	1:1926·5
9^b^	PCR	14·9 months	PRNT_50_ <1:20 DENV 1‐4	1:2243
10^b^	PCR	5·6 months	PRNT_50_ <1:20 DENV 1‐4	1:2946
11^b^	PCR	9·7 months	PRNT_50_ <1:20 DENV 1‐4	1:2811
12^b^	PCR	11·9 months	PRNT_50_ <1:20 DENV 1‐4	1:2519

Note

All donors were naturally infected with ZIKV.

^a^ Infection diagnosed by symptoms, confirmed by PCR and RVP ZIKV neutralization assay. PBMC obtained from NIAID. #1 and #5 are 2 time‐points from the same donor.

^b^ PBMC obtained from OHSU.

### ZIKV reporter virus particle production and neutralization assay

ZIKV (strain H/PF/2013) reporter virus particles (RVPs) were produced as previously described [30]. Briefly, HEK‐293T cells were transfected with plasmids encoding a GFP‐expressing WNV subgenomic replicon and the ZIKV structural genes. To determine viral titre, serial dilutions of RVP‐containing supernatant were used to infect Raji cells that express the flavivirus attachment factor DC‐SIGNR (Raji‐DC‐SIGNR), and GFP‐positive infected cells were quantified by flow cytometry. For neutralization studies, ZIKV RVPs were sufficiently diluted to ensure antibody excess at informative points of the dose–response curve and mixed with serial dilutions of heat‐inactivated human ZIKV convalescent sera for 1 h at 37°, followed by infection of Raji‐DC‐SIGNR cells. GFP‐positive infected cells were quantified by flow cytometry, and results were analysed by non‐linear regression analysis to estimate the dilution of sera required to inhibit 50% of infection (NT50). The reported NT50 values represent the average of two independent neutralization assays, each performed with duplicate technical replicates.

### ELISAs

Approximately 2 × 10^6^ PBMCs were stimulated with 2·5 µg/ml of R848 (Invivogen, San Diego, CA, USA) and 1000 U/ml of IL‐2 (PeproTech, Rocky Hill, NJ, USA) in a 48‐well plate for seven days at 37° and 5% CO_2_. ZIKV NS1 and VLP IgG and the four IgG subclasses (IgG1‐4) were detected in the supernatants and sera by ELISA. Briefly, 96‐well plates were coated overnight with 20 ng/well of ZIKV virus‐like particles (Suriname Z1106033) or 50 ng/ml ZIKV NS1 (Uganda MR766) (Native Antigen Company, Oxfordshire, UK). The plates were blocked for 90 min with 1% BSA. The culture supernatant or diluted sera were added to the wells for 60 min. Plates were washed, and goat anti‐human IgG coupled to HRP (A80‐104P; Bethyl Laboratories, Inc., Montgomery, TX, USA) was added for total ZIKV‐specific IgG detection. HRP‐conjugated secondary Abs for IgG subclass detection include goat anti‐human IgG1 FC (9054‐05), IgG1 hinge (9052‐05), IgG2 FC (9060‐05), IgG3 hinge (9210‐05) and IgG4 FC (9200‐05) (Southern Biotechnology Associates, Birmingham, AL, USA). The assay was developed with TMB substrate (34021; Thermo Scientific, MA, USA), stopped with 1 m HCL and read at 450 nm. For each independent experiment, sera or supernatants were assayed in duplicate and the average OD values obtained presented.

### FluoroSpot assay

FluoroSpot assays were performed as previously described with some modifications [31]. Briefly, IPFL plates (PUV96; CTL, Cleveland, OH, USA) were treated with 20 µl per well 70% ethanol for 1 min. Ig capture antibody (hB2F; CTL), anti‐human IgG (MT91/145; Mabtech, Nacka Strand, Sweden) or anti‐human IgG1 hinge (9054‐05; Southern Biotech) was added to wells, and plates were incubated overnight at 4°. Following washing and blocking of the plates, MBC cultures (2 × 10^5^) were added to duplicate wells for each condition, then incubated overnight at 37° and 5% CO_2_. The plates were washed and blocked with 1% BSA for 1h at 37°. Optimized concentrations of fluorescent (FL) ZIKV NS1 and ZIKV diluted in 100 µl of PBS were then added. The plates were incubated for 1h at room temperature, washed three times with PBS and two times with deionized water, and then rinsed with tap water. The plates were allowed to dry completely. To determine total IgG‐secreting cells, 5 × 10^4^ MBC cultures were added to wells coated with human Ig and visualized using a polyclonal PE‐labelled IgG detection Ab (hB07; CTL).

### Data acquisition

The plates were scanned using an CTL ImmunoSpot S5 Analyzer. Spots representing Abs secreted by antigen‐specific MBCs were detected using different filters (520/40 for DL488 ZIKV or 630/60 for DL594 ZIKV NS1). The settings for the sensitivity of spot counting were established and adjusted manually for each plate using antigen‐stimulated and negative controls to exclude artefacts or background spots. The number of specific or cross‐reactive spots was evaluated by the reader and verified manually [31].

### ZIKV E and NS1 expression on ZIKV‐infected cells

Zika virus FLR strain (GenBank: KX087102) was obtained from BEI Resources (Catalog No. NR‐50183). ZIKV was propagated using the C6/36 cell line. Viral stocks were titrated by ELISPOT using Vero 76 cells. Focus‐forming units (FFU/ml) were determined by immunostaining with anti‐flavivirus antibody 4G2 (Millipore Catalog No. MAB10216). K562 DC‐SIGN cells were infected with ZIKV at a multiplicity of infection (MOI) 0·1 and 1 for 24 h at 37° ± CO_2_. The cells were then washed, resuspended in BD Cytofix/Cytoperm™ (554714; BD Biosciences, San Jose, CA) and incubated for 20 min at 4°. The cells were washed with BD Perm/Wash™ (BD 554723) and stained with mouse anti‐E protein (D1‐4G2‐4‐15) (4G2) (NBP2‐52709) or mouse anti‐ZIKV NS1 antibody (B4) (AbZIKVNS1‐B4, NAC) for 30 min at 4°. Cells were then washed and stained with AF 488 goat anti‐mouse IgG (H + L) (A‐11001). The cells were washed with BD Perm/Wash™ and fixed with BD Cytofix™ (BD 554655). Samples were acquired on a BD LSR™ II flow cytometer, and the data were analysed using the FlowJo software.

### Stable cell line generation

CEM‐NK^R^ cells were obtained from the NIH AIDS Reagent Program and maintained and passaged in RPMI‐1640 supplemented with 10% fetal bovine serum. Cells were transfected with linearized plasmid (pcDNA3.1) expressing a codon‐optimized ZIKV (GenBank Acc# KU681081), DENV‐1 (WestPac74: U88535.1); DENV‐2 (S16681: NC_001474): DENV‐3 (CH53849: DQ863638.1); and DENV‐4 (Singapore /8976/1995: Q5UCB8.1) NS1 proteins designed for maximal surface expression. Stable transfectants were single‐cell‐sorted and selected to obtain high‐level NS1 surface expressing clones.

### Opsonization assay

ZIKV NS1 CEM‐NK^R^ cells (1 × 10^5^) were seeded in a round‐bottom 96‐well plate and incubated with 1:100 and 1:1000 dilution from ZIKV‐immune or ZIKV‐naïve sera for 1h at 4° or 30 min at 37°. Cells were washed and stained with goat anti‐human IgG‐PE (SB 2040‐09). The cells were then washed with BD Perm/Wash™ and fixed with BD Cytofix™ (BD 554655). Samples were acquired on a BD LSR™ II flow cytometer, and the data were analysed using the FlowJo software.

### Human natural killer target visualization assay

The NK‐TVA assay was performed according to the manufacturer's instructions (# NK‐TVA; CTL, Cleveland, OH, USA). Effector cells (cryopreserved PBMC) were thawed, washed and rested overnight at 37° ± 5% CO_2_. Target cells ZIKV NS1 CEM‐NK^R^ cells (1 × 10^6^/ml) were labelled with 1 µl (per ml of cells) of CTL‐TVA fluorescent dye and incubated for 15 min at 37°. After incubation, cells were washed and resuspended, and live cells were counted using the CTL ImmunoSpot S5 Analyzer (CTL). We added 50 µl of target cells (counted with the CTL‐LDC cell‐counting reagent or trypan blue) to a round‐bottom 96‐well plate with 50 µl of different dilutions of sera from ZIKV‐naïve or ZIKV‐immune donors for 1 h at 4° to allow opsonization. Effector cells (PBMC) were counted and added to the culture, and plates were incubated at 37° for 5 h. After incubation, the cells were resuspended, and 50 µl of the cells was transferred in triplicate to a flat‐bottom 96‐well plate. The wells were counted for fluorescent live target cells. The percentage of killing was calculated using the following formula: average count in control wells minus average count in test wells divided by average count in control wells multiplied by 100.

### Intracellular cytokine staining assay

Cryopreserved PBMCs were thawed, washed and rested overnight at 37° ± 5% CO_2_. CEM‐NK^R^‐expressing ZIKV NS1 or DC‐SIGN (negative control) was left in media or incubated with anti‐Zika virus NS1 antibody (B4) or plasma samples at different dilutions for 1 h at 4°. PBMCs were counted, added to the culture and incubated at 37° ± 5% CO_2_ in the presence of mouse anti‐human CD107a (BD 555800) and CD107b (BD 555804). After 1h, BD GolgiPlug™ (BD 555029) and GolgiStop™ (BD 554724) were added to the wells and incubated at 37° ± 5% CO_2_ for another 3 h. Cells were washed and stained with surface Abs anti‐CD3, CD16, CD56 and CD69 for 30 min. Cells were washed, fixed with BD Cytofix™ and acquired on a BD LSR™ II flow cytometer or washed and resuspended in BD Cytofix/Cytoperm™ for 20 min at 4°. The cells were then washed with BD Perm/Wash™ and stained with anti‐IFN‐γ for 30 min at 4°. The cells were washed with BD Perm/Wash™ and fixed with BD Cytofix™. Samples were acquired on a BD LSR™ II flow cytometer, and the data were analysed using the FlowJo software.

### Statistical analysis

All results are reported as absolute mean values plus SEM. IgG subclass distribution, against ZIKV NS1 and VLPs, was compared using the non‐parametric Mann–Whitney test or Friedman test (three or more matched groups) with Dunn's multiple comparisons test. Percentage of lysis of target cells was compared using the non‐parametric Kruskal–Wallis test with Dunn's multiple comparisons test. All statistical analysis was performed using the GraphPad Prism V.9.00 software (San Diego, CA), and a *P* value <0·05 was considered statistically significant.

## RESULTS

### ZIKV NS1‐ and VLP‐specific Abs are predominantly of the IgG1 isotype in immune sera

**FIGURE 1:**
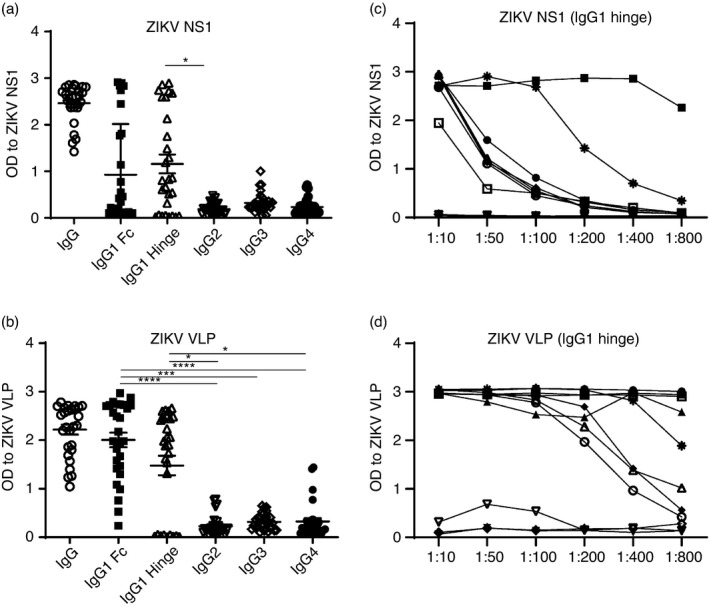
Isotype specificity of ZIKV‐immune sera. ELISA plates were coated with ZIKV NS1 (a, c) or ZIKV VLP (b, d), and a 1:100 dilution (*n* = 28) (a, b) or serial dilutions (*n* = 11) (c, d) of ZIKV‐immune sera were added to plates. Following incubation and washes, optimized concentrations of polyclonal secondary IgG, and monoclonal Abs against the hinge or Fc portion of IgG1, IgG2, IgG3 and IgG4 Abs were added to wells (a) and (b) and IgG1 hinge Abs to (c) and (d). OD values shown at 450 nm. OD values to ZIKV VLP and ZIKV NS1 using a 1:100 dilution of ZIKV‐naïve sera (*n* = 4), <0·2 with polyclonal secondary IgG and <0·1 with IgG1 hinge, IgG1 Fc, IgG2, IgG3 and IgG4 secondary Abs. Bars represent mean with standard error of the mean of the OD values. *P < 0·05, ***P < 0·001 and ****P < 0·0001 were obtained using the non‐parametric Friedman test (three or more matched groups) with Dunn's multiple comparisons test

We determined the magnitude of IgG responses and isotype specificity of ZIKV‐specific Abs in the sera of 28 ZIKV‐immune donors using ZIKV VLPs (to measure gold standard antiviral responses) and recombinant NS1 proteins in ELISAs. We found that sera from all donors had IgG Abs specific to NS1, and to prM and E proteins (present in the VLP preparation). We next used well‐characterized secondary Abs (two IgG1 mAbs directed against either the hinge or the Fc portion, IgG2, IgG3 and IgG4; Southern Biotechnology) to detect the IgG isotype of ZIKV NS1‐ or VLP‐specific Abs. The most commonly detected IgG isotype of NS1 and VLP Abs in ZIKV‐immune sera was IgG1 (Figure 1a,b). Interestingly, we observed a lack of or poor recognition of ZIKV NS1‐ or VLP‐specific Abs in some sera by one or both IgG1 secondary mAbs even though they were all clearly recognized by the polyclonal IgG secondary Abs. Therefore, we asked in a representative subset of sera whether the lack of recognition was unique to ZIKV‐specific Abs or whether Abs to other viruses also followed a similar pattern. We found a poorer recognition of ZIKV NS1 or IAV NP compared with ZIKV VLP Abs in sera from the same individual when the hinge or Fc‐specific secondary Ab was used (Table S1). We next determined whether the magnitude of IgG1 responses to NS1 and VLPs differed and found a stronger Ab response to VLPs overall compared with NS1 (Figure 1c,d).

### ZIKV NS1‐specific memory B cells elicited following natural infection

**FIGURE 2:**
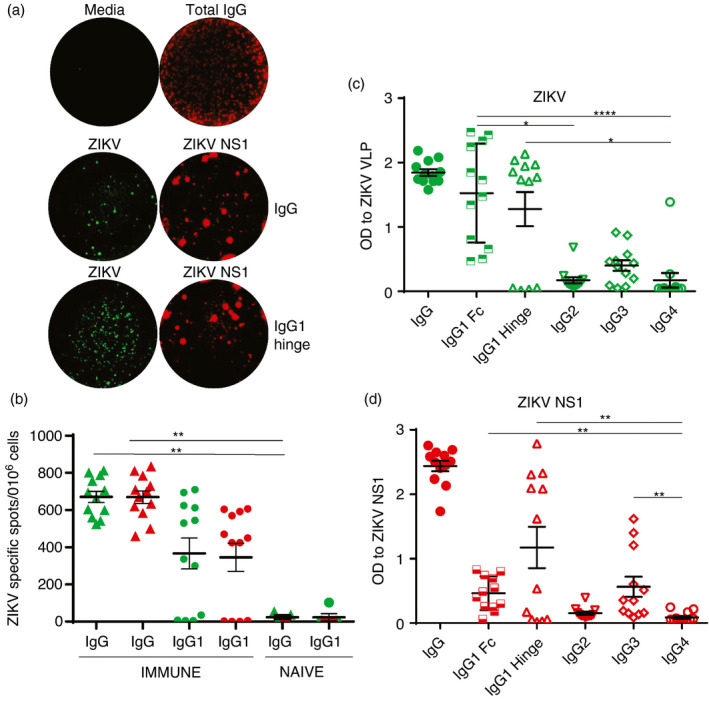
Frequencies of ZIKV‐specific IgG and IgG1 B‐cell responses using fluorescently labelled ZIKV or ZIKV NS1. PBMCs from ZIKV‐immune and ZIKV‐naïve donors were stimulated in vitro for seven days with r848 + rIL‐2. FluoroSpot plates were coated with IgG and IgG1 capture Abs. (a) Images of wells containing media, total IgG, ZIKV and ZIKV NS1 IgG‐ or IgG1‐specific MBCs from one representative donor. (b) Frequencies of ZIKV (green) and NS1‐specific (red) ASCs per 10^6^ input cells in MBC cultures from 12 ZIKV‐immune and three ZIKV‐naïve donors. Values are the mean of duplicate wells for each condition. The supernatants were evaluated for isotype‐specific Abs to (c) ZIKV VLPs and (D) ZIKV NS1. *P < 0·05, **P < 0·01 and ****P < 0·0001 were obtained using the non‐parametric Mann‐Whitney or Friedman test (three or more matched groups) with Dunn's multiple comparisons test

To determine the specificity and magnitude of ZIKV‐specific MBCs, we stimulated PBMC from ZIKV‐immune and ZIKV‐naïve donors with the TLR7/8 agonist, r848, and recombinant (r)IL‐2 in vitro for seven days to convert MBCs into Ab‐secreting cells (ASCs). Time‐points when PBMCs were collected and ZIKV neutralization titres are provided in Table 1. Stimulated cells were added to plates coated with human IgG or IgG1 (hinge) capture Abs. We used optimal concentrations of DL594‐labelled ZIKV NS1 and DL488 infectious ZIKV to identify Abs secreted by ZIKV‐specific MBC cultures using a FluoroSpot assay we recently developed [31]. Representative images of wells containing MBC cultures from a ZIKV‐immune donor with media or total IgG (top panel), ZIKV (green) or ZIKV NS1 (red) with IgG (middle panel) or IgG1 hinge (lower panel) coating Abs are shown in Figure 2a. The frequency of ZIKV and ZIKV NS1 binding ASCs was significantly higher in ZIKV‐immune donors than in ZIKV‐naïve donors (Figure 2b and Table S2). Cell culture supernatants collected 7 days post‐stimulation from immune PBMC also had detectable ZIKV‐ and NS1‐specific Abs. We found a similar pattern of recognition of ZIKV VLP‐ and NS1‐specific Abs by the hinge or Fc‐specific IgG1 secondary Abs in the sera and supernatants from stimulated PBMC in the same donors (Figure 2c,d). We detected no ZIKV VLP or NS1 Abs in supernatants from stimulated cultures of PBMC from naïve donors (data not shown). Taken together, our data indicate robust frequencies of ZIKV NS1‐specific MBCs and predominant secretion of IgG1 ZIKV NS1‐specific Abs by MBCs in the sera of immune individuals.

### ZIKV NS1 monoclonal antibody mediates ADCC

**FIGURE 3:**
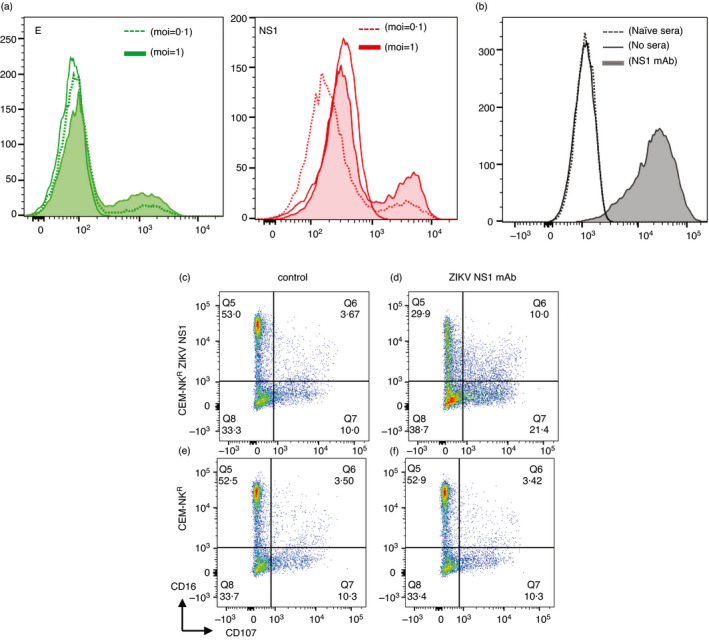
Opsonization and ADCC activity of a ZIKV NS1 monoclonal Ab. (a) K562 cells transfected with DC‐SIGN were infected with ZIKV (moi = 0·1 and 1), and 24 h later, the expression of E and NS1 was measured with monoclonal Abs 4G2 and B4 respectively. (b) Opsonization of ZIKV NS1‐transfected CEM‐NK^R^ cells with a mAb to ZIKV NS1 from Native Antigen Company. CEM‐NK^R^ cells transfected with ZIKV NS1 (c and d) or control CEM‐NK^R^ cells (e and f) were opsonized with a ZIKV NS1 mAb (d and f), added to PBMC. A standard lymphocyte gate based upon light scatter properties followed by selection of singlet viable cells and dump channel exclusion (CD3^−^ T cells) defined the non‐T‐cell lymphocyte population, and Abs to CD16 and CD56 identified NK cells

We first asked whether NS1 was expressed on virally infected cells and found expression of E and NS1 on ZIKV‐infected cells (Figure 3a). To determine whether NS1 Abs could mediate ADCC, we used the CEM‐NK^R^ cell line, which is resistant to NK cell activation in the absence of ADCC. We generated CEM‐NK^R^ cells stably expressing ZIKV NS1 and established assays using a ZIKV NS1‐specific human IgG1 mAb (Native Antigen Company). We first determined whether the ZIKV NS1 mAb could opsonize NS1 on ZIKV NS1‐transfected CEM‐NK^R^ cells, a prerequisite step for ADCC. We found a significant shift in fluorescence in the presence of the NS1 Ab (Figure 3b). We next measured NK cell activation by flow cytometry following a 4‐h incubation of opsonized target cells with healthy PBMC. We found an increase in CD107 degranulation of NK cells only in the presence of the ZIKV NS1‐specific human IgG1 mAb and ZIKV NS1‐transfected cells (Figure 3d) but not control CEM‐NK^R^ (Figure 3f) cells. Furthermore, we saw a clear downregulation of CD16 only in the presence of the ZIKV NS1 Ab (Figure 3d). Conditions in the absence of the NS1 mAb were no different between the ZIKV NS1 and parental cell line (Figure 3c,e).

We optimized and evaluated an image cytometry assay where the number of ZIKV NS1 CEM‐NK^R^ target cells in the presence of the ZIKV NS1 mAb and healthy PBMC as a source of NK cells was measured (Figure S1). We varied the number of ZIKV NS1 target cells (Figure S1A) and the number of effector PBMC (Figure S1B) and evaluated several healthy PBMC for their ability to lyse target cells (Figure S1C). Counts of triplicate wells in the presence of control IgG or ZIKV NS1 mAb are provided (Figure S1D). Based on the loss of calcein dye in dead/dying target cells, we used an established formula to measure % killing in the calcein assay using the average count of target cells in triplicate experimental wells and negative control wells [32]. A decrease in the number of target cells in the presence of the ZIKV NS1 mAb compared with a control IgG Ab was consistently detected (Figure S1).

### ZIKV‐immune sera induces NK cell activation in the presence of ZIKV NS1 target cells

**FIGURE 4:**
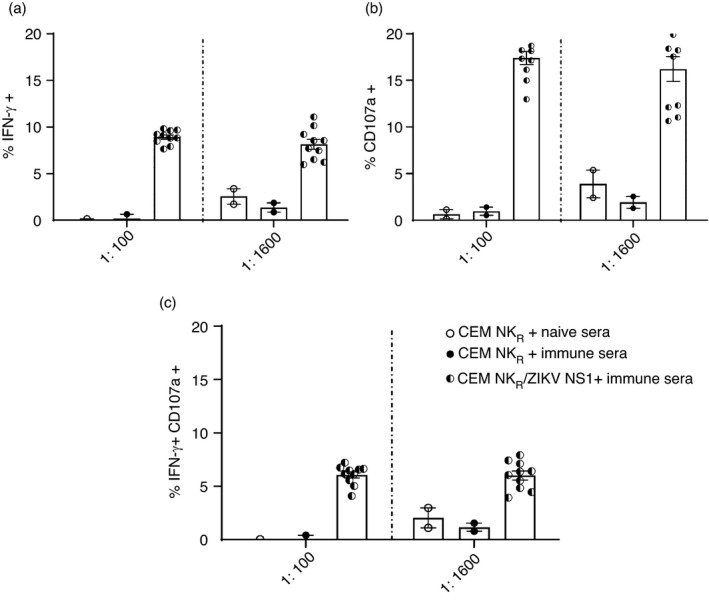
NK cell activation and degranulation in the presence of ZIKV‐immune sera. 1:100 dilution and 1:1600 dilution of polyclonal sera from ZIKV‐immune individuals were evaluated for their ability to mediate ADCC of ZIKV NS1 CEM‐NK^R^ cells using intracellular cytokine assays. Shown are the frequencies of NK cells that (a) secrete IFN‐γ, (B) express CD107 or (C) express both CD107 and IFN‐γ in the presence of ZIKV‐immune sera. Background levels were subtracted

As polyclonal sera contain Abs of varying concentration and function, we next evaluated a performance panel of ZIKV‐immune sera (SeraCare technologies) for their ability to opsonize CEM‐NK^R^ ZIKV NS1 and target cells expressing NS1 from the 4 related DENV serotypes. We found a strong shift in fluorescence with CEM‐NK^R^ ZIKV NS1 and a more moderate shift with the DENV 1–4 NS1 cell lines in the presence of different dilutions of immune sera on DENV‐transfected cells but not untransfected NK^R^ cells (Figure S2). We optimized conditions to assess NK cell activation in the presence of ZIKV‐immune or ZIKV‐naïve sera, healthy PBMC as a source of NK cells and CEM‐NK^R^ ZIKV NS1 target cells. NK cells in PBMC were analysed for CD107a degranulation and IFN‐γ secretion. We detected NK cells expressing IFN‐γ, CD107a and a smaller percentage of NK cells that co‐expressed CD107a and IFN‐γ when ZIKV‐immune sera were added to the assay (Figure 4). Our data indicate that ZIKV NS1 Abs can induce NK cell activation and degranulation.

### ZIKV‐immune sera induce lysis of CEM‐NKR target cells expressing ZIKV NS1

**FIGURE 5:**
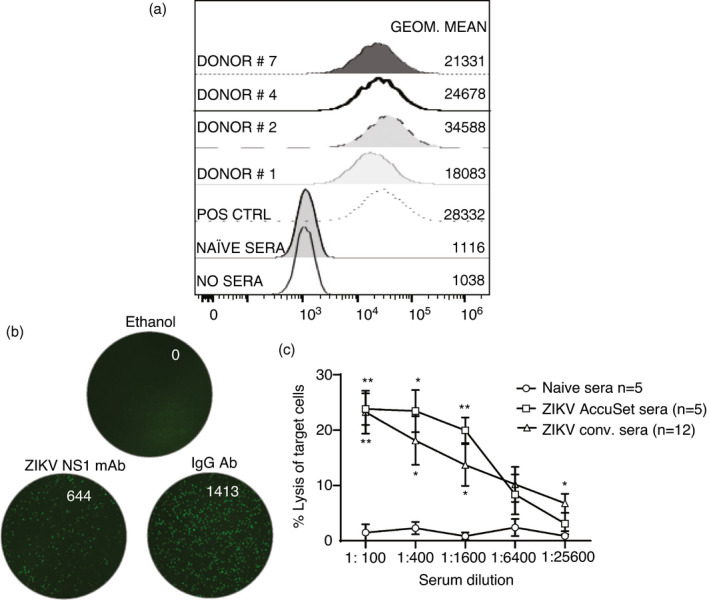
Opsonization and target cell lysis using an NK‐TVA image cytometry assay. (a) Opsonization of ZIKV NS1 CEM‐NK^R^ cells by ZIKV convalescent sera. (b) Representative images of wells acquired by an ImmunoSpot S5 Analyser of an NK‐TVA assay where ethanol, ZIKV NS1 or IgG Ab was added to the wells containing effector and target cells. (c) Using an optimized number of target cells, effector PBMC as a source of NK cells and different dilutions of naïve sera (*n* = 5), positive control sera that opsonized ZIKV NS1 CEM‐NK^R^ targets (*n* = 5) and experimental sera (*n* = 12), the loss of target cells at the single‐cell level was measured by the NK‐TVA image cytometry assay and calculated as a percentage of lysed target cells. *P < 0·05 and **P < 0·01 were obtained using a Kruskal–Wallis test with Dunn's multiple comparisons test

We finally evaluated the ability of ZIKV‐immune sera to opsonize CEM‐NK^R^ ZIKV NS1 cells. A representative subset of immune sera shown in Figure 5a clearly show a shift in fluorescence compared with ZIKV‐naïve sera. We evaluated the ability of ZIKV‐immune sera to induce target cell lysis using the NK‐TVA image cytometry assay. Using an optimized number of target cells, effector PBMC and different dilutions on naïve (*n* = 5), positive control ZIKV‐immune (*n* = 5) and experimental sera (*n* = 12), we measured the loss of target cells at the single‐cell level and calculated the percentage of lysed target cells. Representative images of wells containing ethanol that lyses all cells (positive control), control Ab and the ZIKV NS1 mAb (Figure 5b) are shown. Our results indicate lysis of target cells predominantly in the presence of ZIKV‐immune sera over a range of dilutions (Figure 5c). Taken together, our data demonstrate durable circulating ZIKV NS1 MBC frequencies and serum IgG1 Abs in immune individuals that can utilize ADCC to mediate antiviral function.

## DISCUSSION

Understanding the complex relative contributions of neutralizing and non‐neutralizing Ab effector functions to protection from infection, and clearance of virally infected cells, will likely lead to more effective vaccines. ADCC Ab titres correlate with the control of influenza, HIV and hepatitis C virus infections and with the efficacy of several experimental viral vaccines including influenza and HIV [33, 34, 35, 36, 37] In this study, we demonstrated that ZIKV NS1 Abs elicited following natural ZIKV infection mediate ADCC, as measured by multiple assay formats in vitro.

Many factors contribute to the efficiency of ADCC/Ab‐mediated elimination of virally infected cells: the isotype and glycosylation profile of the Ab; Fc receptor polymorphism; and efficient expression of the antigen on the surface of virally infected cells [33, 39]. Rapid opsonization of the virally infected target cell by Abs compatible with efficient NK cell recognition and activation leads to subsequent destruction of the target cell. Human IgG1 and IgG3 are highly active in NK cell‐mediated ADCC. We found that IgG1 was the dominant subclass of IgG NS1‐ and VLP‐specific Abs in sera and supernatants of stimulated MBCs from ZIKV‐immune individuals, although the magnitude varied in immune donors. We were intrigued by the findings of differential recognition of ZIKV VLP‐, ZIKV NS1‐ or IAV NP‐specific Abs by the hinge or Fc IgG1 secondary Abs. Poor recognition of select antigen‐specific Abs (NS1 but not VLP) by secondary hinge or Fc Abs in individual sera suggests differential glycosylation of Fc receptors rather than Fc receptor polymorphism within an individual as one would expect a consistent lack of recognition of ZIKV VLP vs NS1 vs IAV NP Abs if the latter were true. The pattern of recognition by secondary IgG1 Abs in the sera and MBC supernatants was consistent within an individual. Differential fucosylation of IgG Abs to a closely related flavivirus, DENV, was shown to be a risk factor for thrombocytopenia, a hallmark of severe dengue disease [40]. Whether this holds true for severe ZIKV infections is unknown and should be a focus of future investigation.

We used NS1 fluorescent probes to evaluate the frequency and isotype specificity of MBCs, and determined that IgG1 Abs dominate both the serological and MBC response to ZIKV NS1. We consistently detected larger spots with NS1 probes compared with ZIKV probes and initially speculated that NS1‐specific MBCs secrete more Abs on a per‐cell basis compared with ZIKV MBCs because the size and intensity of spots in each channel relate to the rate of Ab secretion and antibody avidity for each probe [31]. However, as the NS1 protein probe is much smaller compared with ZIKV virions we speculate there is more opportunity for NS1 probes to bind Abs leading to a larger spot size. Our data using other recombinant proteins such as (rE) for dengue viruses support this conclusion (data not shown).

Our previous study demonstrated that the ADCC activity of Abs in plasma obtained before secondary DENV infection inversely correlated with subsequent DENV‐3 plasma viraemia levels and directly correlated with neutralizing Ab titres and anti‐DENV IgG1 levels [38]. However, we did not identify the viral antigen recognized by ADCC antibodies in that study. Abs directed against the E, prM and NS1 proteins of flaviviruses have the potential to mediate ADCC. Assays to measure the ADCC activity of flavivirus Abs have historically depended on the use of the gold standard chromium release assay. High background lysis of target cells and variable CD16 expression on NK effector cell populations in PBMC are well‐known challenges with in vitro assays used to evaluate ADCC activity [32]. We chose CEM‐NK^R^ cell lines expressing ZIKV NS1 to overcome the high background lysis, as they are resistant to NK cell activation in the absence of ADCC. Furthermore, CEM‐NK^R^ ZIKV NS1 cells allow for a clear assessment of the function of NS1‐specific Abs (as opposed to virally infected cells that also express prM and E), provide a consistent timing in the expression of NS1 compared with virally infected cells and are practical and easy to use. In vivo, the expression of NS1 on the surface of virally infected APCs in the circulation and at tissue sites may differ from the expression of NS1 on transfected ZIKV NS1 CEM‐NK^R^ cells. Therefore, despite many advantages, the use of the ZIKV NS1 cell line compared with virally infected antigen‐presenting cells and the in vitro measure of ADCC activity of ZIKV NS1 Abs in the current study have some limitations.

A strength of our study is the evaluation of ZIKV NS1‐specific ADCC Ab activity using two non‐radioactive assays—an intracellular cytokine staining to measure effector NK cell activation and degranulation and an NK‐TVA image cytometry assay to measure the lysis of target cells at the single‐cell level. We chose polyclonal sera with varying amounts of NS1 Abs rather than mAbs and frozen healthy PBMC as a source of effector NK cells rather than an NK cell line expressing high levels of CD16 did to provide a more physiological measure of ADCC activity. Using optimized and validated assays, our data indicate that sera obtained from individuals following natural ZIKV infection have IgG1 Abs to NS1 that mediate ADCC.

Several recent studies isolated and characterized flavivirus NS1‐specific mAbs [22, 24, 25, 41]. Two recent papers demonstrate the potential for flavivirus NS1‐specific mAbs to be evaluated as broad‐spectrum therapeutic Ab targets to treat flavivirus infections [24, 25]. Passive transfer of some of the mAbs protected against lethal challenge in immunocompromised mice and protective mAbs mapped to exposed epitopes in the wing domain and loop face of the β‐platform of ZIKV NS1. Wessel et al. [22] concluded that protection was mediated by complement as many mAbs deposited C3b complement on beads coated with rNS1. Whether these mAbs mediate ADCC is not known.

In summary, we evaluated the isotype specificity and magnitude of ZIKV NS1‐specific Abs and MBCs. Using different assay formats to assess effector cell activation and target cell lysis, we have established that ZIKV NS1 Abs in humans can eliminate ZIKV NS1 target cells via ADCC in vitro. Future studies are needed to define regions on NS1 that are recognized by opsonizing Abs that mediate ADCC. As ZIKV NS1 is highly conserved, NS1 Abs might be able to provide protection to the majority of circulating ZIKV strains unlike DENV NS1 where there is less conservation among the 4 serotypes. Abs that mediate ADCC activity targeting non‐structural proteins may be important for a safe and efficacious ZIKV vaccine.

## CONFLICT OF INTERESTS

The authors have no financial conflicts of interest. Dr. Mathew received reagents from CTL for the NK‐TVA assay.

## AUTHOR CONTRIBUTIONS

LASV, JRC and AM developed the concepts and designed the experiments and wrote the manuscript. LASV, AA, MM, MS and KAD performed experiments and analysed the data. LASV and AM analysed the data and wrote the manuscript. ZLL, TCP and WBM provided reagents, PBMC and sera and provided subject matter expertise. All authors reviewed the manuscript and provided comments.

## ETHICAL APPROVAL

Patient sera and PBMC were collected in accordance with the code of conduct of research with human material. The studies were approved by the institutional review boards. All subjects gave written informed consent.

## DISCLAIMER

Material has been reviewed by the Walter Reed Army Institute of Research. There is no objection to its presentation and/or publication. The opinions or assertions contained herein are the private views of the author, and are not to be construed as official, or as reflecting true views of the Department of the Army, the Department of Defense or the National Institutes of Health. The investigators have adhered to the policies for protection of human subjects as prescribed in AR 70–25.

## Supporting information

Figure S1

Figure S2

Tables S1‐S2
