# Supplementary figures and images for: Non‐structural protein 1‐specific antibodies directed against Zika virus in humans mediate antibody‐dependent cellular cytotoxicity
Source: Immunology. 2021 Jun 14;164(2):386–97. doi: 10.1111/imm.13380 (PMC8442231; doi:10.1111/imm.13380)

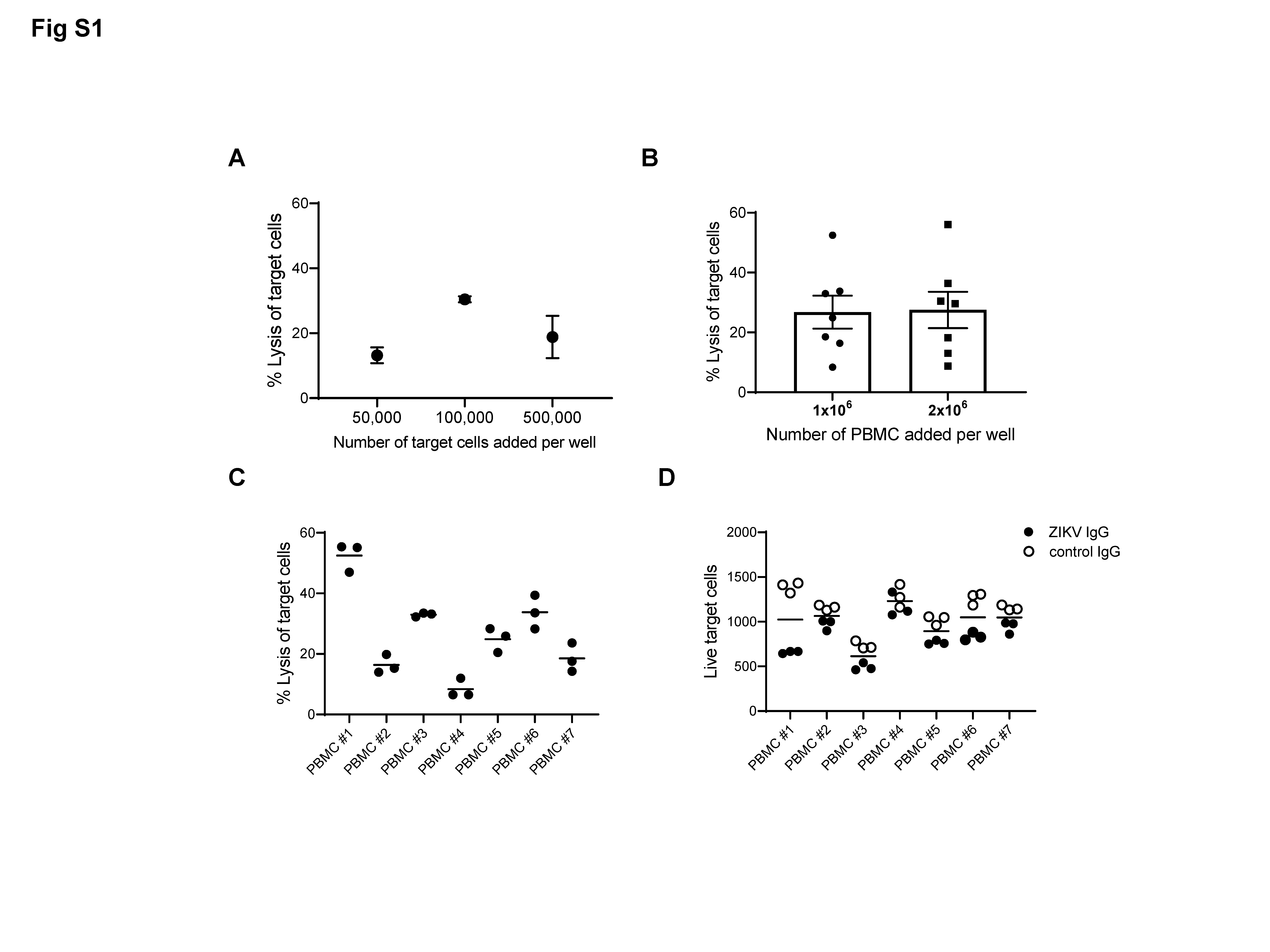

Supplement: Supplementary file 1 — Figure S1 [file IMM-164-386-s001.tif]
