# Supplementary material for: Non‐structural protein 1‐specific antibodies directed against Zika virus in humans mediate antibody‐dependent cellular cytotoxicity
Source: Immunology. 2021 Jun 14;164(2):386–97. doi: 10.1111/imm.13380 (PMC8442231; doi:10.1111/imm.13380)
Supplement: Supplementary file 2 — Figure S2 [file IMM-164-386-s003.pptx]

## Slide 1
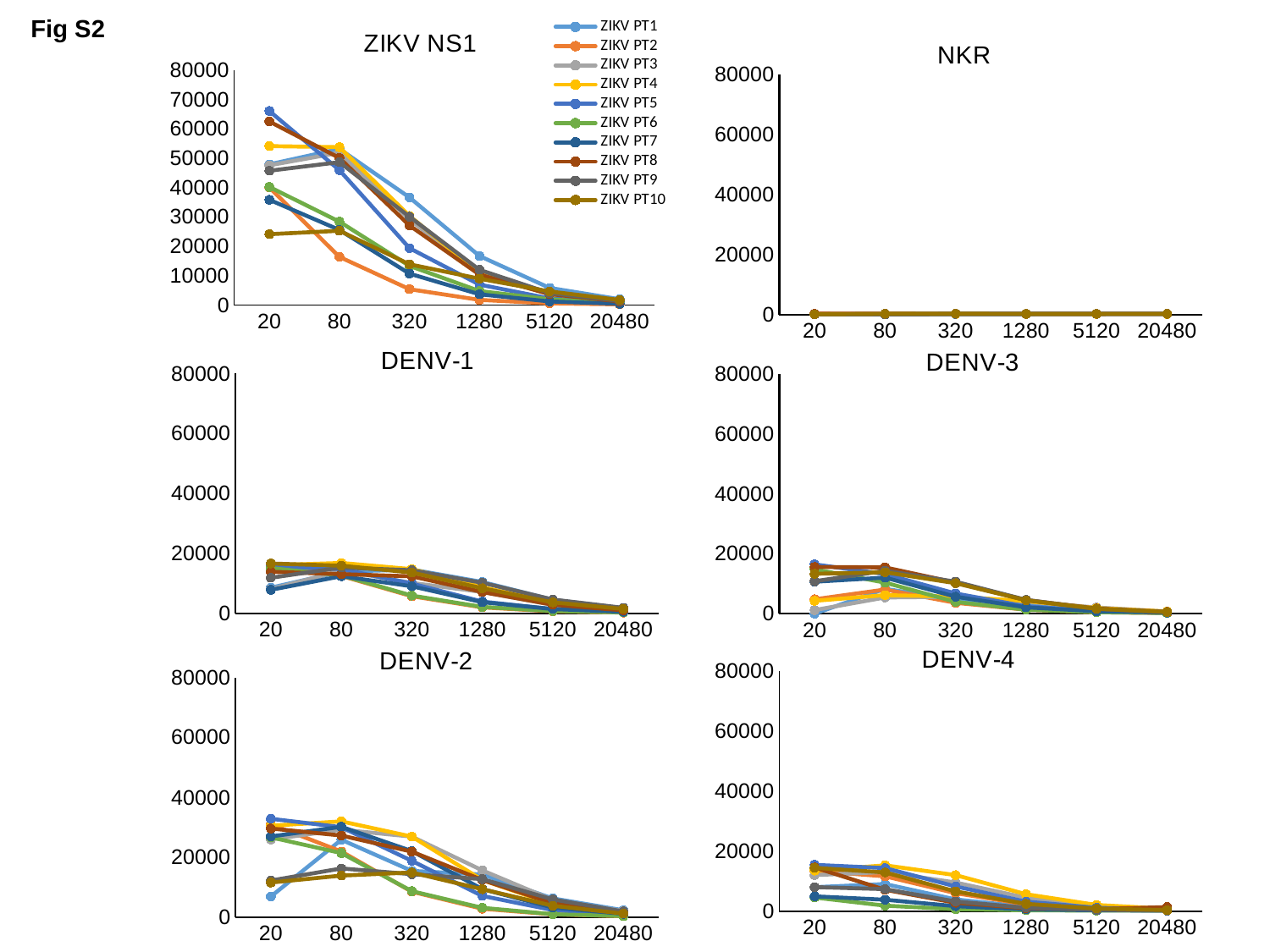

### Chart: NKR
| Category | ZKV PT1 | ZKV PT2 | ZKV PT3 | ZKV PT4 | ZKV PT5 | ZKV PT6 | ZKV PT7 | ZKV PT8 | ZKV PT9 | ZKV PT10 |
|---|---|---|---|---|---|---|---|---|---|---|
| 20 | 30.0 | 237.0 | 30.0 | 30.0 | 30.0 | 210.0 | 30.0 | 241.0 | 30.0 | 62.2 |
| 80 | 120.0 | 208.0 | 30.0 | 30.0 | 158.0 | 206.0 | 30.0 | 238.0 | 30.0 | 241.0 |
| 320 | 141.0 | 187.0 | 183.0 | 182.0 | 210.0 | 210.0 | 209.0 | 227.0 | 200.0 | 259.0 |
| 1280 | 159.0 | 173.0 | 181.0 | 182.0 | 208.0 | 209.0 | 216.0 | 224.0 | 208.0 | 213.0 |
| 5120 | 163.0 | 165.0 | 179.0 | 183.0 | 209.0 | 208.0 | 218.0 | 222.0 | 213.0 | 214.0 |
| 20480 | 162.0 | 163.0 | 173.0 | 180.0 | 207.0 | 209.0 | 219.0 | 217.0 | 225.0 | 226.0 |Fig S2
### Chart: ZIKV NS1
| Category | ZIKV PT1 | ZIKV PT2 | ZIKV PT3 | ZIKV PT4 | ZIKV PT5 | ZIKV PT6 | ZIKV PT7 | ZIKV PT8 | ZIKV PT9 | ZIKV PT10 |
|---|---|---|---|---|---|---|---|---|---|---|
| 20 | 47917.0 | 40045.0 | 47599.0 | 54127.0 | 66071.0 | 40222.0 | 35845.0 | 62511.0 | 45738.0 | 24163.0 |
| 80 | 53176.0 | 16433.0 | 51895.0 | 53768.0 | 45941.0 | 28405.0 | 25596.0 | 50088.0 | 48666.0 | 25314.0 |
| 320 | 36648.0 | 5439.0 | 29170.0 | 30357.0 | 19404.0 | 13373.0 | 10739.0 | 27113.0 | 30089.0 | 13917.0 |
| 1280 | 16727.0 | 1816.0 | 10668.0 | 10859.0 | 7018.0 | 4815.0 | 3707.0 | 10411.0 | 12024.0 | 9015.0 |
| 5120 | 5891.0 | 613.0 | 3799.0 | 3782.0 | 2302.0 | 2007.0 | 1283.0 | 3782.0 | 4142.0 | 4679.0 |
| 20480 | 2029.0 | 294.0 | 1241.0 | 1246.0 | 853.0 | 705.0 | 491.0 | 1291.0 | 1455.0 | 1800.0 |
### Chart: DENV-1
| Category | ZKV PT1 | ZKV PT2 | ZKV PT3 | ZKV PT4 | ZKV PT5 | ZKV PT6 | ZKV PT7 | ZKV PT8 | ZKV PT9 | ZKV PT10 |
|---|---|---|---|---|---|---|---|---|---|---|
| 20 | 8605.0 | 16216.0 | 8123.0 | 16073.0 | 15616.0 | 15139.0 | 7892.0 | 13917.0 | 11891.0 | 16653.0 |
| 80 | 13948.0 | 12513.0 | 14292.0 | 16838.0 | 14775.0 | 12680.0 | 12430.0 | 13109.0 | 15308.0 | 15931.0 |
| 320 | 14645.0 | 5749.0 | 10205.0 | 14775.0 | 9960.0 | 5970.0 | 9075.0 | 12375.0 | 14419.0 | 13642.0 |
| 1280 | 10504.0 | 2047.0 | 7175.0 | 8778.0 | 3971.0 | 2183.0 | 3757.0 | 7159.0 | 10228.0 | 8416.0 |
| 5120 | 4576.0 | 726.0 | 2867.0 | 3484.0 | 1462.0 | 800.0 | 1495.0 | 2918.0 | 4648.0 | 3707.0 |
| 20480 | 1734.0 | 320.0 | 1023.0 | 1332.0 | 551.0 | 361.0 | 582.0 | 1058.0 | 1895.0 | 1525.0 |
### Chart: DENV-3
| Category | ZKV PT1 | ZKV PT2 | ZKV PT3 | ZKV PT4 | ZKV PT5 | ZKV PT6 | ZKV PT7 | ZKV PT8 | ZKV PT9 | ZKV PT10 |
|---|---|---|---|---|---|---|---|---|---|---|
| 20 | 30.0 | 4826.0 | 1216.0 | 4310.0 | 16469.0 | 14808.0 | 10668.0 | 15582.0 | 10835.0 | 13314.0 |
| 80 | 8305.0 | 8069.0 | 5427.0 | 6199.0 | 13196.0 | 10365.0 | 12050.0 | 15444.0 | 14324.0 | 13825.0 |
| 320 | 4648.0 | 3634.0 | 5673.0 | 5698.0 | 6758.0 | 3980.0 | 5673.0 | 10388.0 | 10691.0 | 10183.0 |
| 1280 | 3578.0 | 1353.0 | 4042.0 | 4216.0 | 2472.0 | 1365.0 | 2140.0 | 4556.0 | 4627.0 | 4496.0 |
| 5120 | 1430.0 | 506.0 | 2093.0 | 1874.0 | 867.0 | 507.0 | 840.0 | 1722.0 | 1587.0 | 1633.0 |
| 20480 | 499.0 | 246.0 | 719.0 | 759.0 | 367.0 | 254.0 | 368.0 | 645.0 | 555.0 | 588.0 |
### Chart: DENV-4
| Category | ZKV PT1 | ZKV PT2 | ZKV PT3 | ZKV PT4 | ZKV PT5 | ZKV PT6 | ZKV PT7 | ZKV PT8 | ZKV PT9 | ZKV PT10 |
|---|---|---|---|---|---|---|---|---|---|---|
| 20 | 7998.0 | 13403.0 | 12024.0 | 13522.0 | 15444.0 | 4556.0 | 4956.0 | 14580.0 | 8033.0 | 14451.0 |
| 80 | 9055.0 | 11631.0 | 13051.0 | 15274.0 | 14355.0 | 1845.0 | 3824.0 | 7143.0 | 7352.0 | 12907.0 |
| 320 | 3919.0 | 6010.0 | 9549.0 | 12024.0 | 8250.0 | 667.0 | 1673.0 | 2811.0 | 3246.0 | 6610.0 |
| 1280 | 1455.0 | 2292.0 | 4486.0 | 5661.0 | 3189.0 | 307.0 | 605.0 | 999.0 | 1153.0 | 2444.0 |
| 5120 | 592.0 | 764.0 | 1688.0 | 2075.0 | 1138.0 | 197.0 | 286.0 | 678.0 | 443.0 | 892.0 |
| 20480 | 231.0 | 231.0 | 691.0 | 784.0 | 482.0 | 225.0 | 193.0 | 1368.0 | 240.0 | 360.0 |
### Chart: DENV-2
| Category | ZKV PT1 | ZKV PT2 | ZKV PT3 | ZKV PT4 | ZKV PT5 | ZKV PT6 | ZKV PT7 | ZKV PT8 | ZKV PT9 | ZKV PT10 |
|---|---|---|---|---|---|---|---|---|---|---|
| 20 | 6971.0 | 31037.0 | 25996.0 | 30560.0 | 32878.0 | 26696.0 | 26994.0 | 29626.0 | 12293.0 | 11631.0 |
| 80 | 25881.0 | 21967.0 | 29235.0 | 32015.0 | 30089.0 | 21438.0 | 30223.0 | 27294.0 | 16324.0 | 13948.0 |
| 320 | 15513.0 | 8567.0 | 26934.0 | 26934.0 | 18853.0 | 8759.0 | 22113.0 | 21918.0 | 14419.0 | 14973.0 |
| 1280 | 14292.0 | 2886.0 | 15686.0 | 12485.0 | 7175.0 | 3203.0 | 9444.0 | 12458.0 | 12765.0 | 9402.0 |
| 5120 | 6338.0 | 1039.0 | 5800.0 | 4320.0 | 2461.0 | 1067.0 | 3297.0 | 4741.0 | 5996.0 | 3884.0 |
| 20480 | 2386.0 | 411.0 | 2084.0 | 1462.0 | 840.0 | 437.0 | 1203.0 | 1615.0 | 1998.0 | 1277.0 |
