# Supplementary material for: Non‐structural protein 1‐specific antibodies directed against Zika virus in humans mediate antibody‐dependent cellular cytotoxicity
Source: Immunology. 2021 Jun 14;164(2):386–97. doi: 10.1111/imm.13380 (PMC8442231; doi:10.1111/imm.13380)
Supplement: Supplementary file 3 — Tables S1‐S2 [file IMM-164-386-s002.pdf]

Table S1: IgG1 Ab responses to ZIKV VLP, ZIKV NS1 or IAV NP

Donor ID	ZIKV VLP			ZIKV NS1			IAV NP		
	IgG	IgG1 Fc	IgG1 Hinge	IgG	IgG1 Fc	IgG1 Hinge	IgG	IgG1 Fc	IgG1 Hinge
1	2.70	1.67	2.49	2.71	0.12	0.83	1.65	0.07	0.18
2	2.61	1.95	2.24	2.52	0.11	0.66	1.43	0.07	0.25
3	2.63	2.72	2.49	2.69	0.13	1.49	1.86	0.07	0.52
4	2.57	0.76	2.07	2.59	0.12	0.52	1.79	0.05	0.11
5	2.63	2.73	2.41	2.44	0.08	0.54	1.98	0.06	0.18
6	2.65	1.98	0.03	2.37	0.08	0.02	1.58	0.04	0.02
7	2.50	2.50	2.88	2.81	2.81	0.12	1.62	0.08	0.03
8	2.68	2.68	2.74	2.43	2.43	2.74	2.57	0.23	1.61
9	2.80	2.80	0.06	2.72	0.10	0.04	2.17	0.08	0.04
10	1.58	1.58	0.03	2.82	0.34	0.31	2.31	0.07	0.03
11	2.17	2.17	2.60	2.86	0.44	2.86	2.41	0.28	1.49
12	1.83	1.83	2.44	2.84	0.42	1.29	2.48	0.06	0.27

A 1:100 dilution of sera from donors naturally infected with ZIKV was used in ELISA binding assays.

Table S2: Summary of ZIKV NS1 and ZIKV MBC frequencies

Donor ID.	Approximate days after infection	Total ASCs/10^6 input cell	Number of ZIKV-Specific MBCs			
			ZIKV spots		ZIKV NS1 spots	
			IgG	IgG1	IgG	IgG1
1[#]	138 days	15280	757.5	695	635	470
2[#]	52 days	10900	812.5	545	700	425
3[#]	68 days	14920	525	710	730	605
4[#]	66 days	12520	695	625	710	592.5
5[#]	14 days	10160	560	530	835	570
6[#]	43 days	10200	787.5	5	810	0
7[#]	24 days	NT	600	35	500	5
8[#]	41 days	NT	605	300	585	450
9*	1 year	10500	665	7.5	670	0
10*	1 year	11200	542.5	5	620	2.5
11*	1 year	8860	707.5	612.5	785	607.5
12*	1 year	10340	802	337	460	425
Naïve 1	-	8920	37.5	5	12.5	2.5
Naïve 2	-	15020	55	102.5	55	102.5

All donors were naturally infected with ZIKV.

[#] Infection diagnosed by symptoms, confirmed by PCR and RVP ZIKV neutralization assay.

[#]PBMC obtained from NIAID. * PBMC obtained from OHSU.

NT = not tested.
